# Twisted Intramolecular Charge Transfer (TICT) Controlled by Dimerization: An Overlooked Piece of the TICT Puzzle

**DOI:** 10.1021/acs.jpca.1c00629

**Published:** 2021-04-05

**Authors:** Ahmed M. El-Zohry, Esam A. Orabi, Martin Karlsson, Burkhard Zietz

**Affiliations:** † Department of Chemistry − Ångström Laboratory, Uppsala University, Box 523, SE-75120 Uppsala, Sweden; ‡ Department of Physics − AlbaNova Universitetscentrum, Stockholm University, SE-10691 Stockholm, Sweden; § Department of Chemistry, University of Manitoba, Winnipeg, Manitoba R3T 2N2, Canada; ∥ Applied Physical Chemistry, KTH Royal Institute of Technology, Teknikringen 30, SE-10044 Stockholm, Sweden

## Abstract

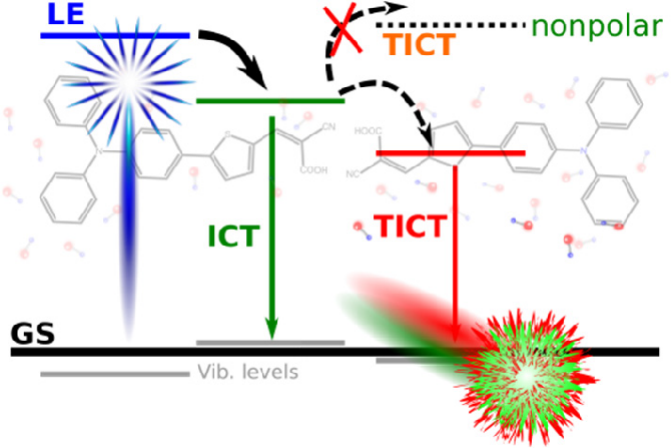

Organic dyes have shown high efficiencies in solar cells, which is mainly attributed to the push–pull strategy present in such dyes upon attaching to the semiconductor surfaces. We deeply studied the fundamental photophysical properties of cyanoacrylic dyes, mostly the L1 dye, and found unique emission properties that depend on many factors such as the solvent polarity and the concentration of the dye and could present a complete emission picture about this family of dyes. The L1 dye shows an intramolecular charge transfer (ICT) emission state at low concentrations (approximately nanomolar scale) and shows a twisted intramolecular charge transfer (TICT) emission state in specific solvents upon increasing the concentration to the micromolar scale. Moreover, the associated emission lifetimes of the ICT and TICT states of the L1 dye depend on solvent basicity, highlighting the role of hydrogen bond formation on controlling such states. Density functional theory calculations are performed to gain insight into the photophysical properties of the dye and revealed that H-bonding between the carboxylic groups triggers the dimerization at low concentrations. Using femtosecond transient absorption, we assigned the rate of TICT formation to be in the range (160–650 fs)^−1^, depending on the size of the studied cyanoacrylic dye. Therefore, we add herein a new dimension for controlling the formation of the TICT state, in addition to the solvent polarity and acceptor strength parameters. These findings are not limited to the studied dyes, and we expect that numerous organic carboxylic acids dyes show similar properties.

## Introduction

**Figure 1:**
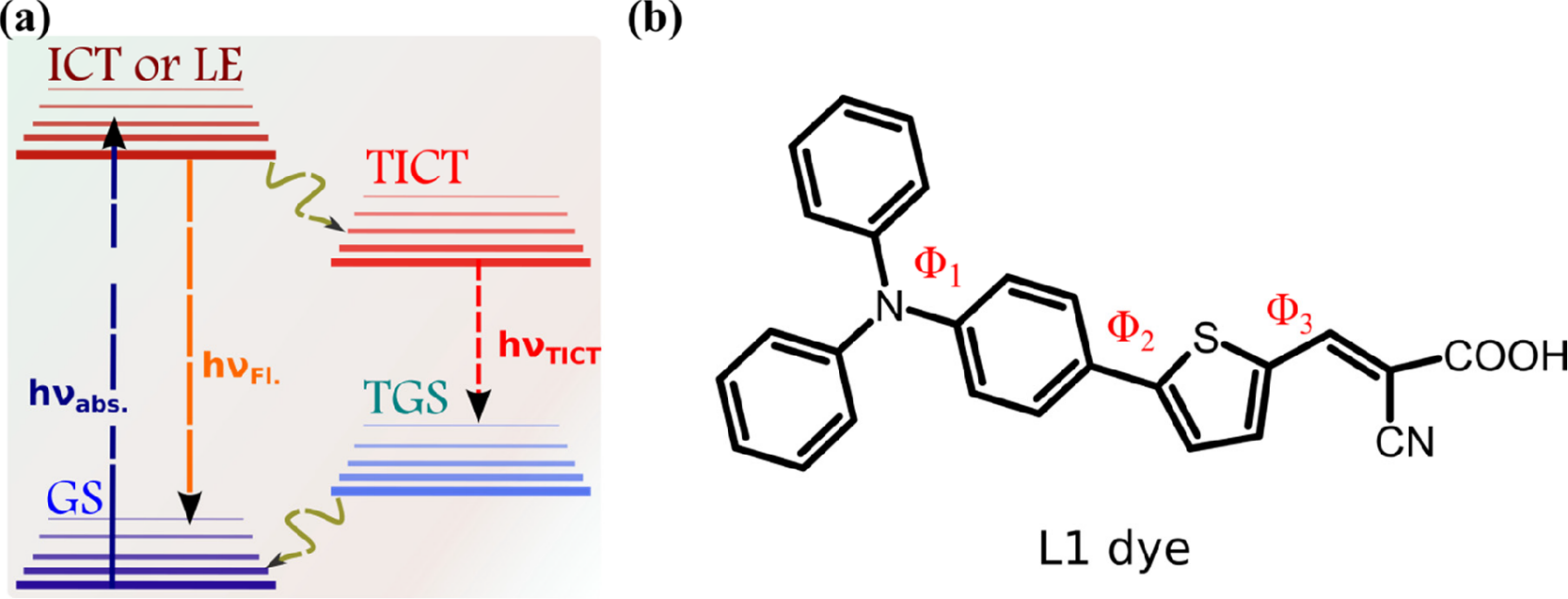
(a) Schematic energy diagram of the TICT state formation populated from LE or ICT state (GS: ground state; TGS: twisted ground state). (b) Chemical structure of the L1 dye, with three main labeled dihedral angles (see the text for more information).

Numerous donor–acceptor substituted aromatic compounds show dual fluorescence, as first observed by Lippert et al.,^1^ and is explained by the twisting of the donor relative to the acceptor group.^2−5^ The twisting process implies a decoupling of the two groups, i.e., donor and acceptor, and leads to considerable charge transfer and a highly polar, charge-separated state known as the twisted intramolecular charge transfer (TICT) state.^3,6^ This state is formed after populating the Franck–Condon locally excited (LE) state^2^ (see Figure 1a). Twisting around a single bond in the excited state can then lower the molecule’s energy, forming the TICT state. From there, the electronic relaxation can occur with emission of fluorescence of a large Stokes shift, leading to a twisted electronic ground state that quickly converts to the relaxed form. Several conditions need to be fulfilled to form the TICT state. The donor and acceptor groups have to be of sufficiently high polarity to allow for an efficient charge separation, and a polar environment is needed to stabilize the formed polar state. Therefore, TICT emission is usually only seen in polar solvents. Twisting involves a decoupling of the π-systems of the donor and acceptor units, as is typical for a biradicaloid system. The transition dipole strength between the ground and excited state for such a system is expected to be very small. Besides being a fundamental photophysical phenomenon, TICT has found applications that include pH and ion indicators,^7^ high selectivity for specific ions,^5^ fluorescent probes,^8^ liquid crystals,^9,10^ fluorescent solar collectors,^4,11^ and volume sensing in polymers.^12^ Recently, it was shown that the TICT state stabilizes the charge separation in dye-sensitized solar cells (DSSCs) through small charge recombination rate.^4,13−16^ For solar cell applications, a charge transfer process is designed to move from the donor to the acceptor moiety, forming an intramolecular charge transfer (ICT) state,^13^ which is considered as a primary emissive state. Thus, the ICT state herein corresponds to the LE state in the traditional studied TICT molecules (see Figure 1a).^1^

Herein, we primarily report on a new red-shifted emission from the L1 dye (5-[4-(diphenylamino)phenyl]thiophene-2-cyanoacrylic acid) upon concentration variation in various solvents. This dye was designed as a photosensitizer in DSSCs and comprises a triphenylamine as a primary donor and the cyanoacrylic acid (CyA) as a primary acceptor and anchor group (see Figure 1b). Remarkably, the red emission is only observed at higher concentrations, micromolar scale, and in polar solvents and is assigned to the TICT emission from L1 dimers. Noticeably, the emission lifetimes of the TICT state are shorter than the ICT state even if the dimers are present. Density functional theory (DFT) calculations confirm the stability of dimers in solution and the changes in the potential energy surface (PES) at certain dihedral angles of the L1 dye, which support the experimental observations about large-scale motions of the dye in the excited state. Earlier observations of red-shifted emission from dimers of the classical TICT molecule (dimethylamino)benzonitrile (DMABN) and from self-complexes in supersonic jets have been attributed to excimers and not to a TICT state.^17^ The presence of TICT process of cyanoacrylic dyes in solution confirms the previous finding about the role of TICT process to minimize the charge recombination in solar cells.^4,16,18,19^ This is thus the first account of dimer TICT emission for cyanoacrylic dyes in solution, which as well can be extended to other organic dyes with similar acceptor moieties.

## Methods

### Chemicals

The L1 dye (5-[4-(diphenylamino)phenyl]thiophene-2-cyanoacrylic acid (CAS 762269-56-7) was obtained from Dyenamo AB. However, the synthesis of L1 was described earlier.^20^

### Steady-State Absorption and Emission

Absorption and emission spectra were performed at a right angle in a 1 cm cuvette by using a Varian Cary 5000 and an Horiba Jobin Yvon Fluorolog, respectively. Emission measurements were automatically corrected for wavelength-dependent instrument sensitivity.

### Time-Correlated Single Photon Counting

The TCSPC setup has been described in detail previously.^21^ Briefly, the sample was excited with a picosecond diode laser (Edinburgh Instruments, EPL405) at 404.6 nm (77.1 ps pulses), giving an instrument response function of ca. 100 ps.

### Streak Camera Measurement

The streak camera setup has been described earlier.^21^ Briefly, excitation of the sample with ultrafast laser pulses was performed by using a frequency-doubled Ti:Sa oscillator (Coherent Mira) output (400 nm). Fluorescence at a right angle to the excitation was passed through a Bruker SPEC 250IS spectrograph and onto the streak camera (Hamamatsu streak camera and blanking unit C5680 in combination with a Synchroscan Unit M5675). The charge-coupled device (CCD) camera (Hamamatsu Orca-ER C4742-95) was used in binning mode (2 × 2 pixels) to give a 512 × 512 pixel matrix.

### Transient Absorption

A detailed description of the transient absorption setup can be found in a previous publication.^22^ Briefly, the ca. 100 fs (1 kHz) output pulses from a Coherent Legend amplifier were split, and one part was frequency doubled for 400 nm excitation. Another part of the output was sent into a moving CaF_2_ plate to produce white light as the probe beam. After passing through the sample and a spectrograph, the light was detected from ∼390 nm to up to 700 nm by a 512 pixel photodiode array.

### Computational Details

Quantum chemical calculations are performed to gain insight into the change of the absorption and emission spectra of L1 in response to its charge (neutral, protonated, and deprotonated) and molecular aggregation (monomer vs dimer). The geometry of L1 and its deprotonated and various protonated forms was optimized in the ground (S_0_) and first excited state (S_1_), and the absorption and emission spectra were calculated with the time-dependent DFT method.^23^ These calculations were performed in acetonitrile, described implicitly by using the polarized continuum model (PCM), with the Gaussian 16 program.^24^ Density functional theory (DFT) calculations were performed with the 6-31G(d) basis set and the CAM-B3LYP functional.^25^ This functional is chosen as it takes long-range interactions into account, which is important to describe ICT states.^26^ Relaxed potential energy scans of the dihedral angles ϕ_1_, ϕ_2_, and ϕ_3_ of L1 (see Figure 1b) were performed in acetonitrile in the S_0_ and S_1_ states to explore the impact of excitation on molecular twisting and natural bond orbital (NBO) analysis was performed to quantify the degree of intramolecular charge transfer in the two states. Intermolecular interactions and its impact on the absorption spectra of L1 were investigated by using the (L1)_2_ dimer as a model. The S_0_ geometry of the (L1)_2_ dimer was optimized in the gas phase starting from various initial binding conformations, and the interaction energies of all stable structures (no imaginary frequencies) are calculated^27−29^ without (*E*) and with (*E*^CP^) correction for basis set superposition error (BSSE) by using the counterpoise procedure of Boys and Bernardi.^30^

## Results and Discussion

**Figure 2:**
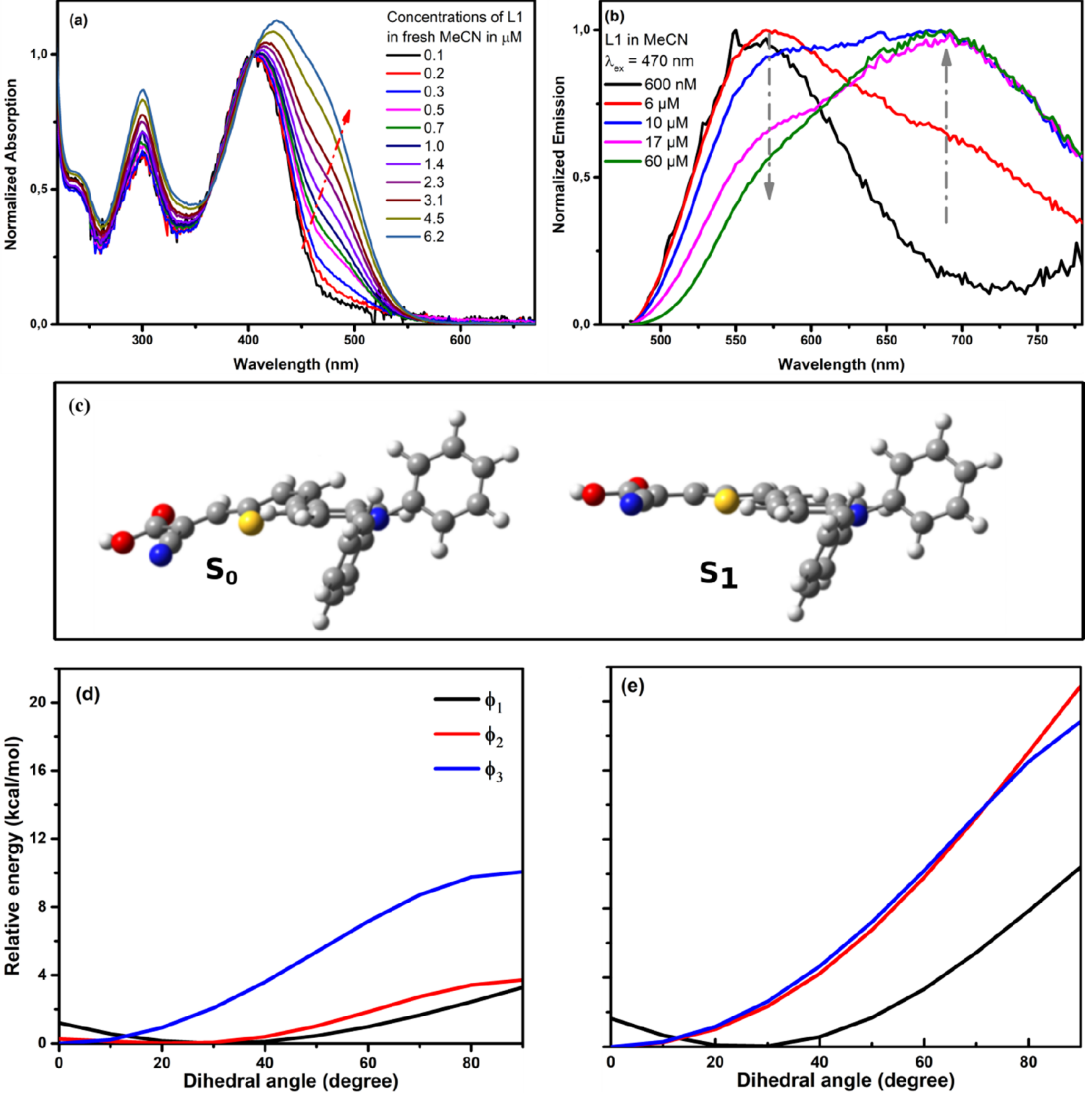
Normalized absorption (a) and emission (b) spectra of the L1 dye in acetonitrile. (c) Global minimum structure of L1 in the S_0_ and S_1_ states calculated with CAM-B3LYP/6-31G(d) in acetonitrile. Atom color code: H (white), C (gray), N (blue), O (red), and S (yellow). Potential energy curves calculated from relaxed scan of ϕ_1_ (black), ϕ_2_ (red), and ϕ_3_ (blue) of L1 in the S_0_ (d) and S_1_ (e) states.

The normalized absorption and emission spectra of L1 in acetonitrile are shown in Figure 2a,b at various concentrations on the micromolar scale. The maxima of the lowest absorption and emission bands, corresponding to S_0_ and S_1_ at low concentration of L1, are at ca. 405 and 570 nm, respectively. The absorption band is red-shifted, and a new shoulder appears at ca. 470 nm; correspondingly, a new emission band appears at ca. 700 nm when the concentration of L1 is increased (Figure 2a,b). The global minimum structures calculated with CAM-B3LYP/6-31G(d) in acetonitrile for L1 in the S_0_ and S_1_ states are reported in Figure 2c. In the S_0_ state, L1 is characterized by a dipole moment (μ) of 7.3 D and by 30°, 26°, and 0° for ϕ_1_, ϕ_2_, and ϕ_3_, respectively. In comparison, L1 is more polar (μ = 12 D) and more planar (ϕ_1_ = 26°, ϕ_2_ = 0°, and ϕ_3_ = 0°) in the S_1_ state. NBO analysis reveals charges of 0.17 and 0.28 for the triphenylamine moiety in the S_0_ and S_1_ states, respectively. This indicates that ICT occurs to a greater extent in the excited state, which is consistent with the larger polarity of the S_1_ vs S_0_ state. This ICT is responsible for the observed trigonal planar rather than triagonal-pyramidal geometry of the triphenylamine moiety (Figure 2c). The potential energy curves calculated from relaxed scans of ϕ_1_, ϕ_2_, and ϕ_3_ of L1 in the S_0_ and S_1_ states are shown in Figure 2d,e. Besides a switch in ϕ_2_ from 26° to 0° upon excitation, results show much larger energy barriers for rotations in the excited state. This indicates that structural rearrangements happen before relaxation of the excited L1 molecules.

The new spectral bands that appear upon increasing the L1 concentration are assigned here to the dimers formed between L1 molecules through the CyA groups according to the following simple equation:
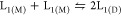
1in which M stands for monomer and D stands for dimer. The dimerization constant based on the absorption changes at 525 nm by using the modified Benesi–Hildebrand equation^31^ is ca. 9.0 × 10^5^ M^–1^ (see the fitting in Figure S1). Such a high association constant is matching with the low concentration utilized for such observations.^32^ These spectral changes are not only limited to the L1 dye but are also observed in other cyanoacrylic dyes with different molecular weights such as the D131 and L0 dyes (see Figures S2 and S3).

**Figure 3:**
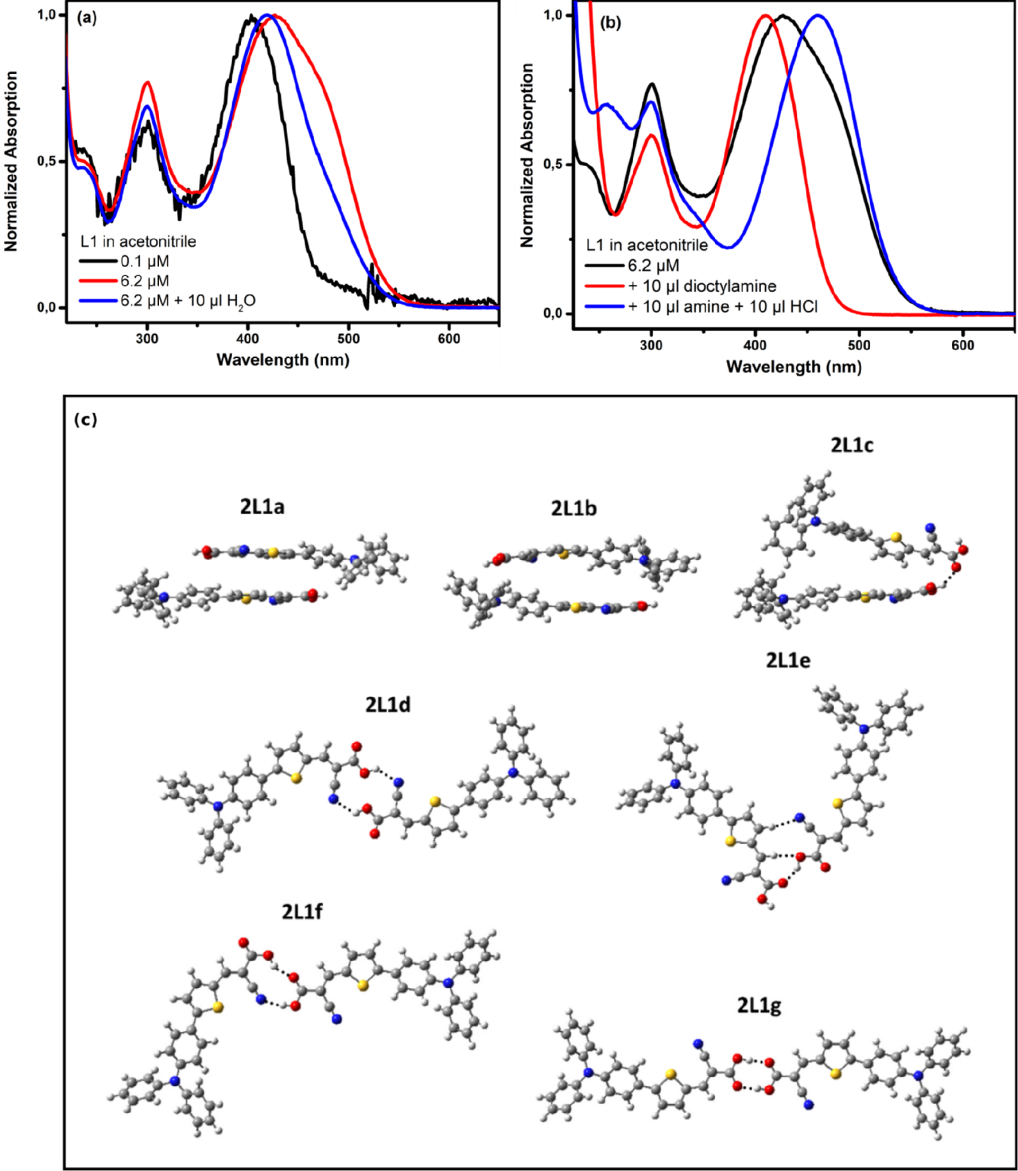
Addition of water (a) and the octylamine base (b) to a solution of L1 in acetonitrile. Addition of HCl is also shown after adding the base to the L1 solution. (c) Optimized minimum-energy structures of the (L1)_2_ dimer. Structures are arranged from top left to bottom right in order of increasing the interaction energy. Dotted lines connect H-bonded atoms. Atom color code: H (white), C (gray), N (blue), O (red), and S (yellow).

We have excluded the possibility of L1 deprotonation at low concentrations by the possible traces of water present in aprotic MeCN by measuring the absorption spectra of L1 in the presence of water and in EtOH (pure protic solvents) (see Figure 3a and Figure S4). Figure 3a shows the absorption spectra of L1 at moderate concentrations of 6 μM, in which two absorption maxima are detected; the addition of 10 μM of water minimizes slightly the band at 470 nm, without complete disappearance. This experiment shows that protic solvents such as water can only disturb the dimer formation.

Interestingly, the equilibrium between monomers and dimers of L1 can be shifted as well by only adding organic bases (Figure 3b) and acids such as HCl or carboxylic acids (see Figure 1b and Figure S5). The addition of the dioctylamine base is expected to deprotonate the L1, which inhibits the formation of the dimers, giving an absorption spectrum similar to the monomer form of L1 (Figure 3b). The following addition of excess HCl to the basic solution of L1 and the recall of L1’s absorption at the dimer position confirms the stability and the reversibility of such an equilibrium dimerization reaction of L1 (see Figure 1b). The formation of L1 dimers can happen through the carboxylic acid group solely or through the CN group as well. Previously, the crystal structure of 2-CyA showed hydrogen-bonded dimers through the cyano group,^33^ which were also suggested by infrared measurements for a similar CyA-containing dyes.^34^ However, addition of carboxylic acids such as acetic acid to L1 results in absorption spectra similar to that of the L1 dimer (Figure S5). In addition, measuring the L0Br dye (a derivative of L0 with Br instead of CN) yields similar absorption behavior to that of L0 (see Figure S6). These evidences preclude the role of the CN group in the dimerization process itself.

The structures of the various stable conformers of (2L1) calculated with CAM-B3LYP/6-31G(d) in the gas phase are given in Figure 3c, and their interaction energies are reported in Table 1. Structures with stacking arrangements of the molecules (**2L1a**–**2L1c**) are less stable than those with planar orientation of the interacting pair (**2L1d**–**2L1g**), indicating that dimerization is attributed to H-bonding rather than hydrophobic association of the aromatic groups. The most stable structure (**2L1g**) is stabilized by two (O–H···O=C) H-bonds between the carboxylic groups and display an interaction energy *E*^CP^ = −20.7 kcal/mol. Structure **2L1f** is 6.3 kcal/mol less stable than **2L1g**, confirming that dimerization is derived by the carboxylic rather than the cyano group.

**Table 1:** Interaction Energy (kcal/mol) of the Structures Presented in Figure 3^a^

structure	*E*	*E*^CP^
**2L1a**	–6.9	–1.1
**2L1b**	–11.9	–4.1
**2L1c**	–12.7	–7.2
**2L1d**	–11.2	–8.7
**2L1e**	–13.7	–11.0
**2L1f**	–17.6	–14.4
**2L1g**	–24.8	–20.7

a *E* and *E*^CP^ are interaction energies without and with correction for BSSE.

**Figure 4:**
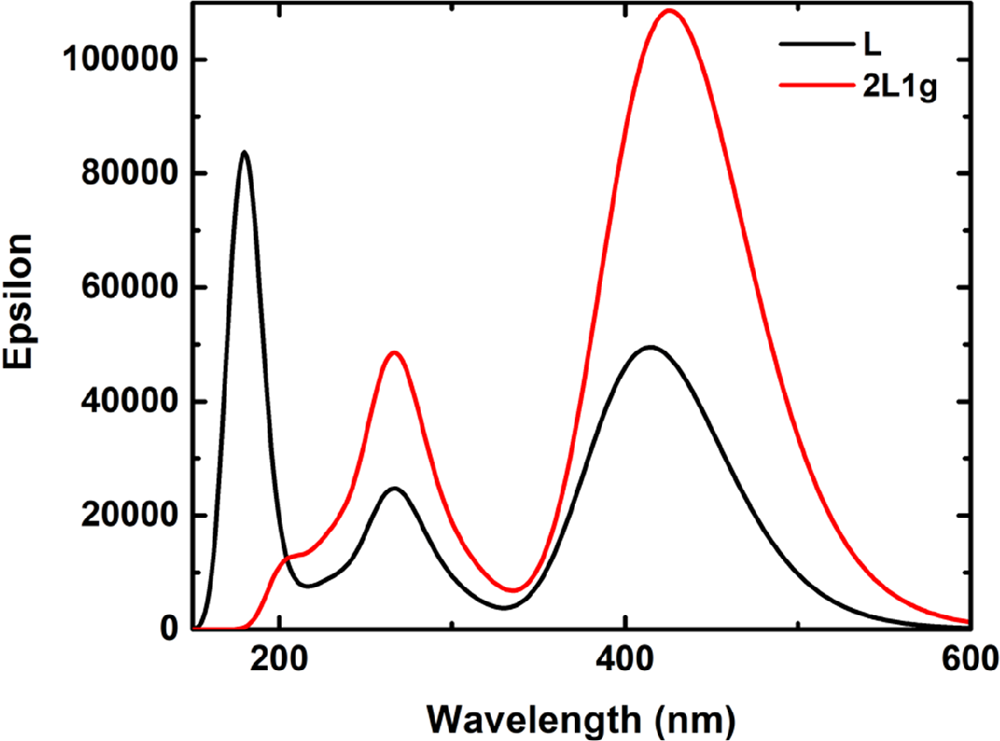
Calculated absorption spectra of L1 and **2L1g** in acetonitrile.

The geometry of the most stable conformer (**2L1g**) was also optimized in acetonitrile, and its absorption spectra were calculated (see Figure 4). Results show a slight red-shift in λ_max_ relative to this of the isolated monomer (429 vs 415 nm), confirming that the observed increase in λ_max_ with increasing the concentration of L1 (Figure 2a) is a consequence of dimerization.

With regard to the emission data of L1, we assign the emission at 570 nm to the ICT state, in which the charge is transferred from the triphenylamine to the CyA moiety as shown previously by TD-DFT.^4,13,35−37^ However, we attribute the red emission at ca. 700 nm to a new emission from the TICT state. Because of the low concentration utilized (micromolar) and the polarity of the environment, we excluded the formation of exciplex or excimers.

**Figure 5:**
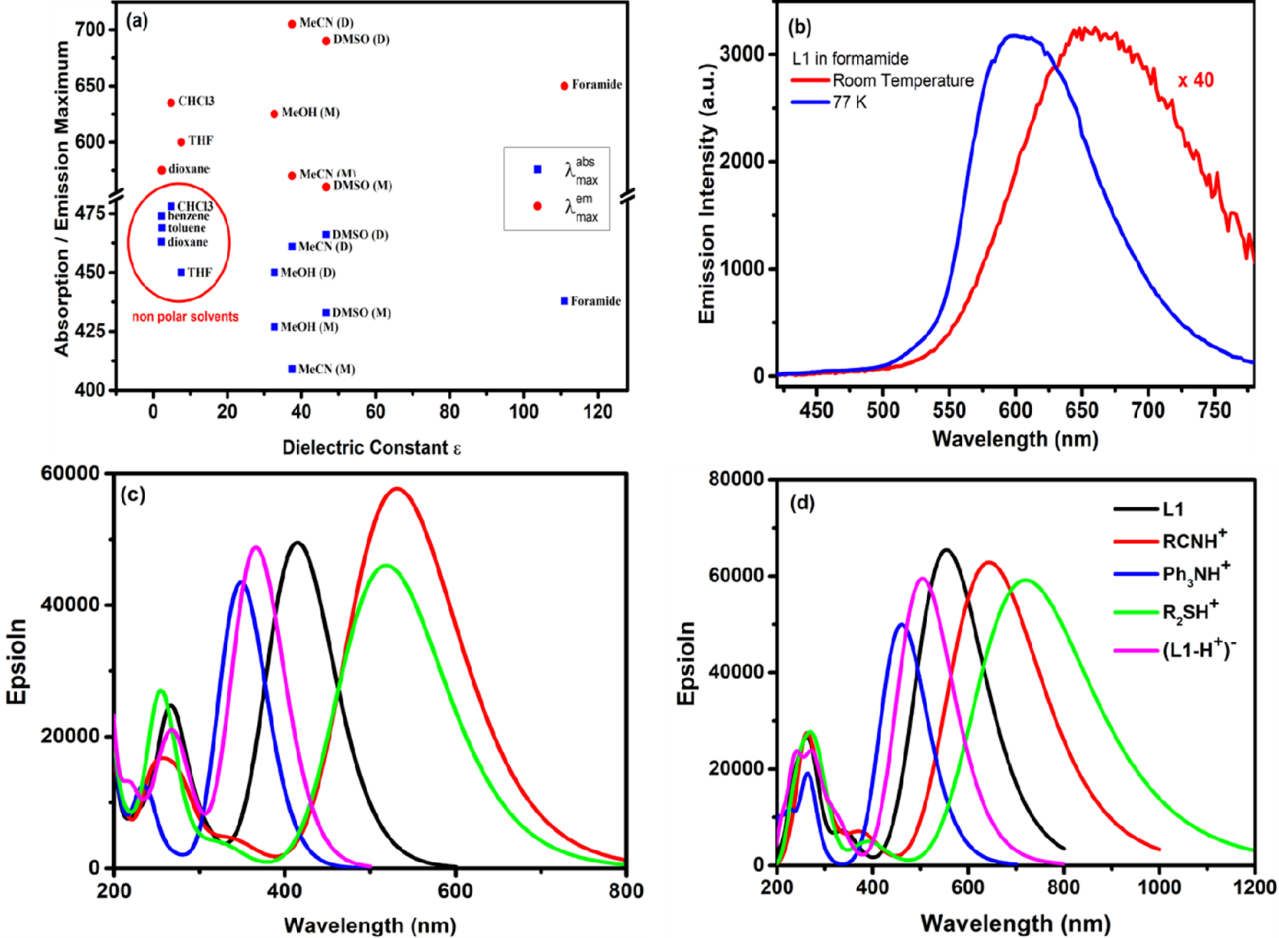
(a) Absorption and emission maxima of L1 in different solvents; M represents monomers, and D represents dimers. (b) L1 emission in formamide at room temperature and at 77 K (400 nm excitation). The emission centered at 675 nm (TICT) is disabled in the solid state; instead, the normal emission completely dominates (ICT). (c) Calculated absorption and emission (d) spectra of L_1_ in the neutral (black), protonated (RCNH^+^ (red), Ph_3_NH^+^ (blue), R_2_SH^+^ (green), and deprotonated form (L1-H^+^)^−^.

We have also measured the L1 dye in a wide range of solvents with different properties to correlate between the monomer–dimer equilibrium and the solvent effect on the ICT and TICT states. Figure 5a shows the absorption and emission maxima for L1 in various solvents of different properties by using dielectric constant herein. As can be seen, in polar solvents, L1 can have different absorption and emission maxima depending on the utilized concentration. At low concentration, in which monomers are expected to be the dominant species, more blue absorption and emission maxima were measured, while at high concentrations of L1 more red spectra were measured representing the dominant dimer state of L1. Simultaneously, the emission lifetime measurements were performed, and different lifetimes associated with different emissive states were found (Figure 5a and Table 3). Interestingly, only the absorption band centered at 675 nm was seen in formamide (ε ≈ 115), regardless of L1’s concentration. This behavior can be explained by complex formation between L1 monomers and the highly polar formamide molecules, which have been confirmed by the titration in CHCl_3_. Apparently, a highly stable complex species can be formed between formamide and L1 dye (see Figure S7). This reflects the tendency of the cyanoacrylic group to interact with the solvent molecules through hydrogen bonding.^32,38^ Despite, the absence of monomer–dimer equilibria of L1 in formamide, the formamide complex species show as well an emission–temperature relationship reflecting the presence of an actively excited state process, as shown in Figure 5b. The emission of L1 in formamide, at low temperature (77 K), shows an emission increase of 40 times, with a significant blue-shift of the emission band (to 600 nm), which has been attributed to the restriction of large-scale motions in the excited state, i.e., the inhibition of the TICT state (Figure 5b).

On the other hand, in nonpolar solvents (CHCl_3_, dioxane, tetrahydrofuran, toluene, and benzene), no changes in absorption and emission bands were observed upon changes in concentration; however, L1 is expected to be in dimeric form (see Figure 5a for absorption and emission maxima in a range of solvents). Thus, L1 is assumed to exist as dimers in nonpolar solvents, as the positions of the absorption maxima of L1 are close to those of the dimers in polar solvents. Also, nonpolar solvents are expected to interact less with the polar cyanoacrylic groups in L1 and are thus less likely to break the dimers. The formation of dimers in CH_3_Cl has been confirmed by comparing the absorption spectra after adding a carboxylic acid and an organic base to the solution of L1 in CH_3_Cl (see Figure S8). The absorption spectrum of L1 in CHCl_3_ is slightly changed after adding the carboxylic acid but significantly changed upon adding the organic base, which confirms the presence of the L1 molecules in the dimer form in nonpolar solvents. Thus, Figure 5a shows that L1 predominantly exists in the dimeric form in solvents with ε < 20 but exists in both monomeric and dimeric forms in solvents with 20 < ε < 60. Monomers are, however, the main species in solvents with ε > 100 due to interaction between L1 monomers and the solvent molecules.

As stated previously, adding organic bases or acids to soluble L1 shows similar effects to the monomer–dimer equilibrium in various solvents. To investigate the impact of strong acids and bases on the absorption and emission spectra of L1, we calculated the absorption and emission spectra of the protonated and deprotonated forms of L1 in MeCN. We consider the S and two N atoms as possible protonation sites in L1. The geometry of these three protonated forms and the geometry of the deprotonated molecule (carboxylate ion) were optimized in the S_0_ and S_1_ states. The calculated absorption and emission spectra of the L_1_, (L1+H^+^)^+^, and (L1–H^+^)^−^ species are reported in Figure 5c,d. Calculations predict a maximum absorption wavelength of the neutral dye (L1) at 415 nm, in very good agreement with the experimental value (405 nm). The fact that the absorption spectrum of L1 in the presence of HCl is red-shifted relative to this of the neutral dye (462 vs 405 nm, Figure 2d) indicates that protonation does not occurs at the triphenylamine N atom, as this will inhibit the ICT process. In fact, calculations predict that such protonated form has a λ_max_ = 349 nm. In line with experiment, the RCNH^+^ and R_2_SH^+^ protonated forms show red-shifted absorption spectra (λ_max_ = 531 and 519 nm, respectively). However, the lower basicity of the R_2_S vs RCN group and the fact that the R_2_SH^+^ structure is 24 kcal/mol less stable than RCNH^+^ indicate that the latter is the protonation form of L1 in the presence of strong acids such as HCl and TFA. Similar to experiment, calculations predicts a blue-shifted absorption of the deprotonated species (λ_max_ = 366 nm) relative to the neutral molecule. The fact that calculations overestimate and underestimate λ_max_ of the protonated and deprotonated species, respectively, is likely due to the implicit description of the solvent. Charge transfer from and toward the solvent molecules surrounding the protonated and deprotonated species would improve the agreement with the experiment. Calculations predict an emission spectrum of L1 with λ_max_ = 555 nm, in a very good agreement with the experimental value (570 nm). In comparison, the RCNH^+^ protonated form displays emission spectra with λ_max_ = 645 nm, while the acetate anion possesses λ_max_ = 505 nm. The ϕ_1_, ϕ_2_, and ϕ_3_ dihedral angles for the RCNH^+^ protonated form in the S_0_ state are (18°, 5°, 0°) and are (29°, 5°, 0°) in the S_1_ state. As can be seen, the calculations provide more accurate results for the protonated form than the pure L1 dimers (see Figure 4); thus, we correlate the behavior of dimers in solution to the RCNH^+^ protonated form, as the calculated spectra of the latter provide similar results to the experimental ones. The relaxed excited state of the protonated form is 10 kcal/mol less than the corresponding relaxed state of the neutral form (see Figure S9). This indicates that the TICT state of the L1 dimer will be more stable than the ICT state of the L1 monomer.

**Figure 6:**
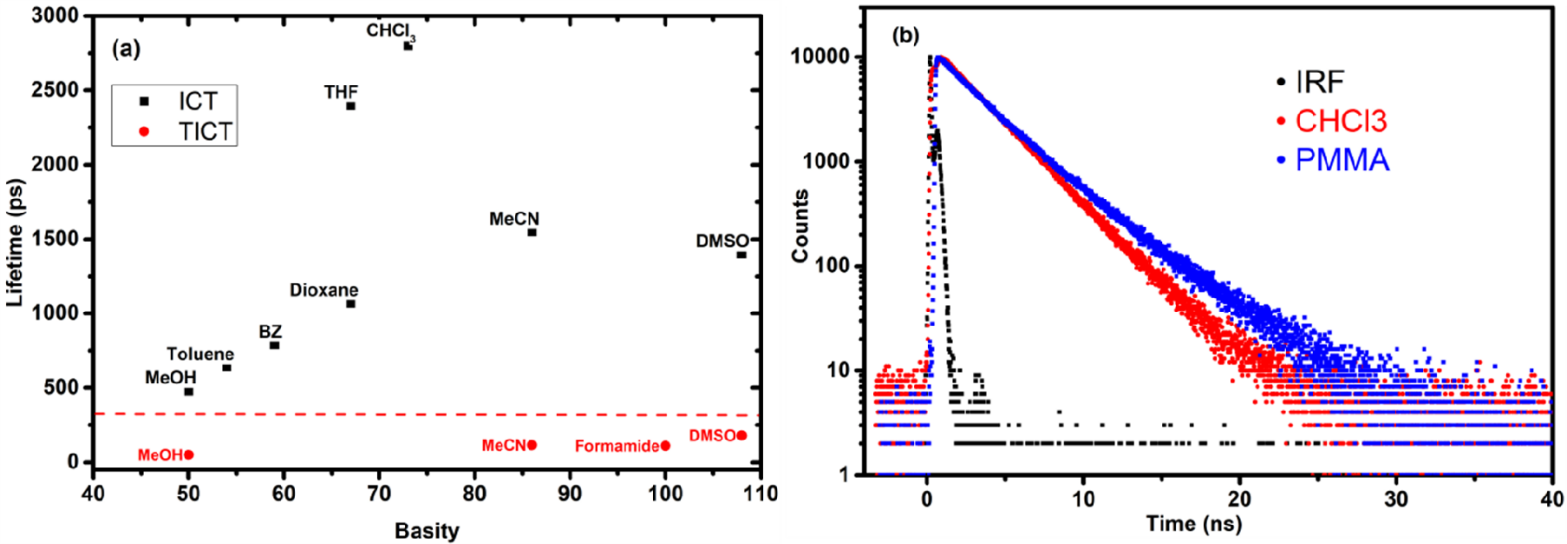
(a) Lifetime of the ICT and TICT states for L1 vs the solvents’ basicity. The dotted red line corresponds to the difference between TICT and ICT states. (b) Fluorescence emission intensity of L1 in CHCl_3_ and PMMA versus time.

The time-resolved emission data were measured with a streak camera and time-correlated single photon counting (TCSPC) to further characterize the two emission bands. The ICT and the TICT bands were found to be solvent-dependent, showing emission lifetimes of 0.5–3.0 ns for the ICT state and ca. 50–200 ps for the TICT state (Figure 6a and Table 2). The emission lifetimes of L1 were measured in different solvents at low and high concentrations, and only in polar solvents, except formamide, did the emission lifetimes differ between high and low concentrations. The lifetimes in a wide range of solvents are plotted against the basicity^39^ of the solvent in Figure 6a, showing a nice correlation between the emission lifetimes and solvent basicity. The solvent basicity reflects the solvent property to accept hydrogen bonds.^40,41^ Clearly, the large scale motions of L1, in either ICT or TICT, depend on the H-bond interactions in the excited state. Similar solvent effects have been detected previously but a carboxylic acid dye.^32^ As can be seen, only at high concentrations (dimers) and in sufficiently polar solvents, TICT is enabled as a faster deactivation channel. In nonpolar solvents, such as benzene, the L1 is expected to be in the dimeric form; however, the lifetime measurements are independent of concentration, and the dimers in these nonpolar solvents show long-lived ICT emission. To check the suppression of TICT state in L1, we measured the embedded form of L1 in the PMMA matrix (poly(methyl methacrylate)), and as expected long-lived emission species with lifetime of ca. 3.5 ns (81%) and 1.2 ns (16%) were measured. The minor 1.2 ns component is likely to represent L1 in a slightly different environment, possibly interacting with specific groups of the PMMA.^39^ Comparing the lifetime emission of L1 embedded in PMMA and in CH_3_Cl confirms the inability of dimers in nonpolar solvents to populate efficiently the TICT state. The 3.5 ns component is assumed to be close to the radiative lifetime of L1 and a sign of a complete inhibition of the TICT formation. Similarly, the TICT formation could be completely prevented in frozen formamide by lowering the temperature to 77 K (see Figure 5b). These measurements also highlight the fast conversion between LE and ICT state in L1, as the stiff conditions in PMMA could not prevent the population of ICT state. Thus, our mixing herein between the LE state and the ICT state is plausible.

**Table 2:** Lifetimes (in picoseconds) for the ICT and TICT States of L1 in Different Solvents

solvent	ICT	TICT
MeCN	1550	100
MeOH	450	50^a^
DMSO	1400	175
formamide		110
dioxane	1100	
benzene	790	
toluene	650	
THF	2400	
CHCl_3_	2800	
PMMA	3500	

a The TICT lifetime in MeOH is below the instrument response function of the SPC setup.

**Figure 7:**
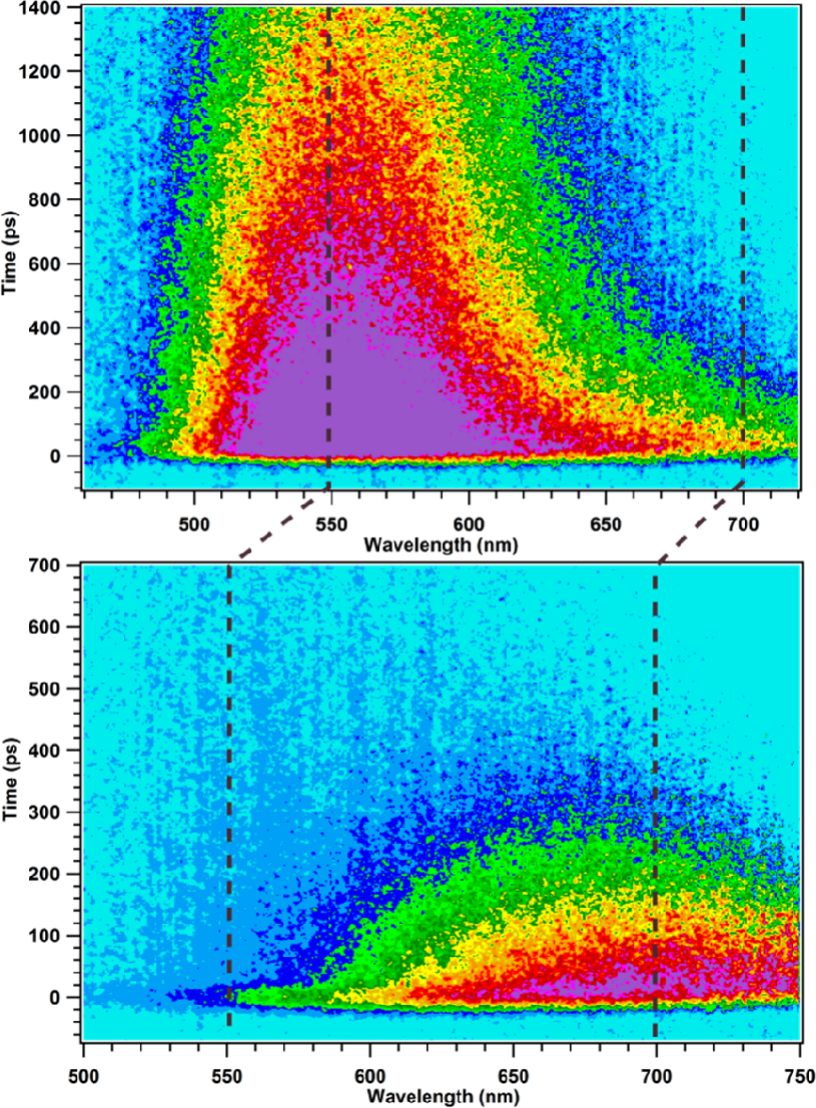
2D-false emission plot of L1 in DMSO at 200 nM (top) and 20 μM (bottom). Mind the different time scales in ps. The dotted black lines indicate to the emission spectral shifts due to the emission states; check the text for more information.

To monitor the spectral changes of emission for L1, the transient emission of L1 in DMSO is shown herein, as it shows the most striking spectral changes with variation of concentration. At low concentration (monomer case), the main emission centered at 550 nm is assigned primarily to the ICT state of monomers (Figure 7, top). However, the emission is not homogeneous, and an emission tail is clearly visible between ca. 650 and 720 nm. Kinetic traces were fitted at 700 nm, and a lifetime of ca. 215 ps was obtained. At 550 nm, two lifetimes of ca. 300 ps and 1.4 ns were obtained (the latter from TCSPC). We assign the 200–300 ps component to red-shifted emission from the TICT state. At high concentration, the ICT emission band has nearly completely vanished, and the TICT emission band is centered on 650 nm (see Figure 7, bottom). Fitting by a global procedure gave two lifetimes, ca. 3 ps for the solvent dynamics of DMSO^42^ and 175 ps for the decay of the TICT state.

**Figure 8:**
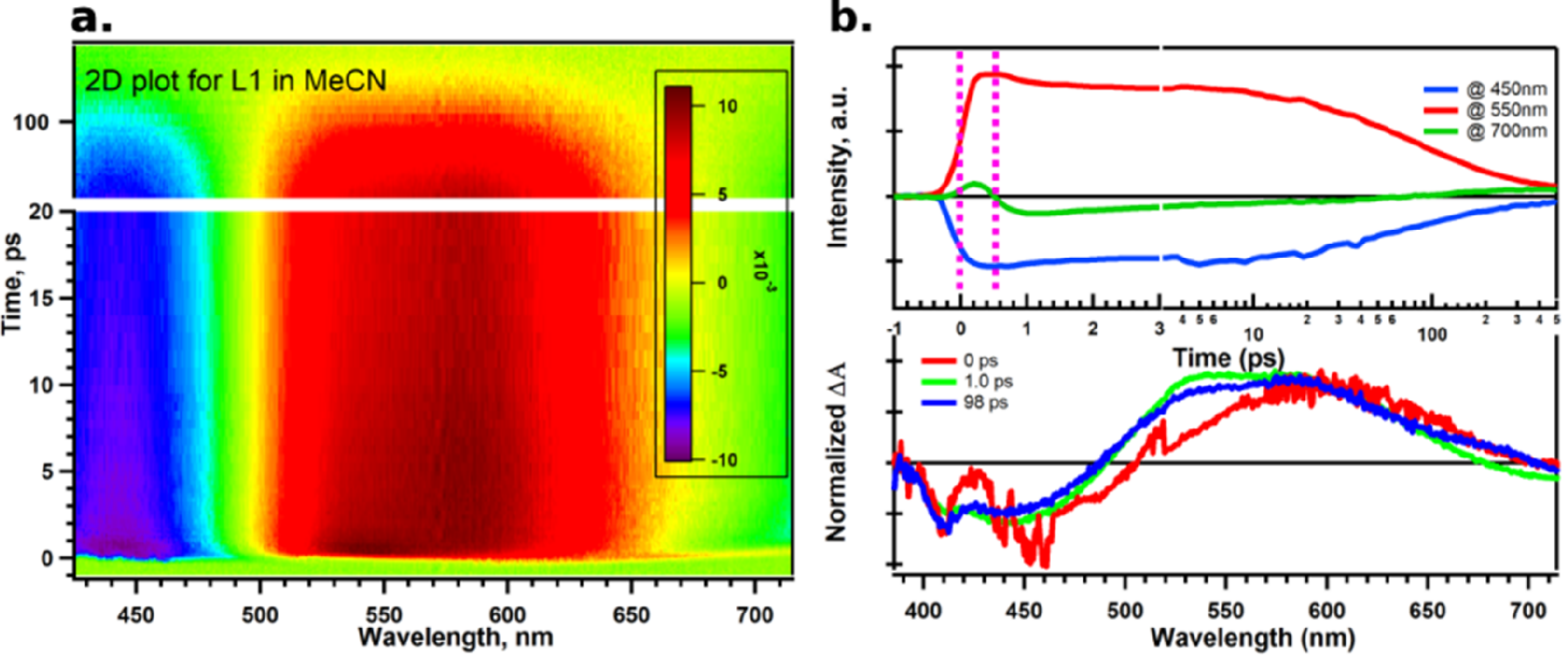
(a) Difference absorption of L1 in acetonitrile after excitation at 400 nm, 2D-color plot. (b) Extracted kinetics and spectra. The two dotted pink colors show the delay of the emission related to the ESA due to the TICT state formation. See text for more information.

Femtosecond transient absorption spectroscopy (TA) was used to characterize the transition between the initially excited ICT state and the TICT state according to the scheme shown in Figure 1. The results for L1 in acetonitrile at high concentration (dimers) after excitation at 400 nm are seen in Figure 8a,b. The ground state bleach (GSB) in the region around 450 nm could be fitted with two lifetimes of 1.6 and 97 ps, while for the regions dominated by excited-state absorption (ESA, 550 nm) and 700 nm (ESA + stimulated emission), three lifetimes are needed in the fits, including an additional fast component of 160–350 fs. This fast lifetime component, which is only visible in the excited state and not the GSB, is attributed to the transformation of the ICT state to the TICT state, which then shows stimulated emission at long wavelengths (ca. 700 nm). This fast lifetime is also associated with spectral blue shifts in the ESA region from 600 nm to ca. 525 nm. The lifetimes at different wavelengths are summarized in Table 3. Similar observations were found for the TA of D131 dye (another cyanoacrylic dye) in MeCN at high concentrations. After the population of the ICT state of D131 dye, a blue-shift in the ESA was observed due to the formation of the TICT state with a formation lifetime of ca. 650 fs (Figure S10). The larger formation lifetime in D131 than in L1 can be assigned to the larger molecular size of D131. Similar correlations between the dye molecule size and such large-scale motions have been discussed previously.^15^

**Table 3:** Lifetimes for L1 in Methanol from Fits of Kinetic Curves at Given Wavelengths

	lifetimes (amplitudes)		
wavelength (nm)	τ_1_ (ps)	τ_2_ (ps)	τ_3_ (ps)
450		1.6 (−10%)	97 (−90%)
550	0.16 (−58%)	0.68 (22%)	98 (30%)
700	0.35 (64%)	1 (−25%)	92 (−11%)

**Scheme 1:**
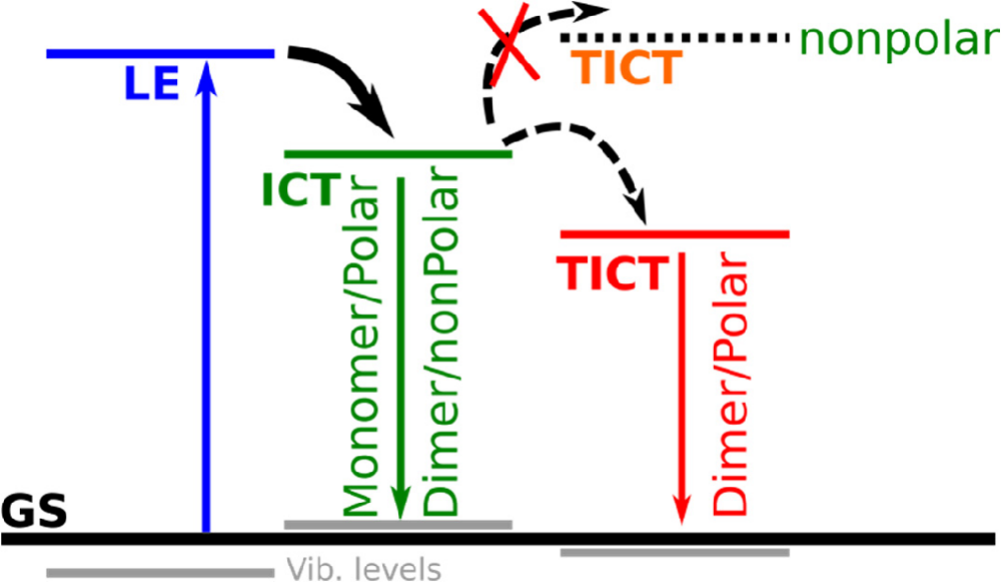
Schematic Representation for the Reaction Pathways in the Excited State of L1 upon Changing Solvent Polarity along with the Presence of Monomer Dimer Species in the Solution

The observations from absorption and fluorescence spectroscopy as well as from DFT calculations are summarized in Scheme 1. Upon excitation of the cyanoacrylic dye (L1), the LE state directly populates the ICT that is normally the emissive state; the TICT state is not accessible for the monomer of L1 and for dimers in nonpolar solvents due to an energy barrier present. However, the dimer formation in polar solvents as well as protonation increases the acceptor strength and leads to a lowering of the energy of the TICT state relative to the ICT state, which becomes the lowest emission state, which is characterized by spectral and kinetic changes in the emission properties. Thus, through fine-tuning of the L1 concentration, both emissive states can be detected.

It is worth mentioning to compare between the nature of TICT for L1 in solution and on mesoporous surfaces. We previously interpreted that presence of the TICT state in the L1 on semiconductor surfaces hinders the charge recombination process,^4,14^ indicating that the TICT state should have long-lived emission that the ICT state. This apparent contradiction can be understood via the degree of freedom that the molecules have in solution and not on semiconductor surfaces or in the single crystals. In either way, it is clear now that further increase of the acceptor strength of the cyanoacrylic group by dimerization in polar solvents or by attaching it to a semiconductor surface will facilitate the population of the TICT state, leading to red emission in polar solvents and minimize the charge recombination in solar cells.

## Conclusion

We have studied the photophysical properties of cyanoacrylic dyes in solution taking the L1 dye as model for this class of organic dyes utilized primarily in solar cells as photosensitizers. Our study revealed the presence of monomer/dimer equilibrium with high association constant. The dimerization has been confirmed experimentally and theoretically to be through the carboxylic acid group. The formation of the L1 dimer modifies the strength of the acceptor group and thereby shifts the energy level of the two lowest excited states (ICT and TICT) in polar solvents such that TICT is stabilized sufficiently to become the lowest and fluorescent state. This opens possibilities for tuning the emission across the visible spectrum solely by changes in dye’s concentration. Also, we could correlate the high performance of cyanoacrylic dyes in solar cells due to the population of the TICT state through increasing the acceptor strength of the cyanoacrylic dye.^4,14^ Thus, our study contributes to both fundamental and applicable understandings for such organic dyes.

## Supporting Information Available

Figures S1–S10: the presence of monomer–dimer equilibrium for other cyanoacrylic dyes, the effect of adding acids or bases on the absorption and emission data, as well as the fs-TA for the D131 dye in MeCN showing the conversion form the ICT to the TICT state (PDF)
