# Supplementary material for: Twisted Intramolecular Charge Transfer (TICT) Controlled by Dimerization: An Overlooked Piece of the TICT Puzzle
Source: J Phys Chem A. 2021 Apr 5;125(14):2885–94. doi: 10.1021/acs.jpca.1c00629 (PMC8154600; doi:10.1021/acs.jpca.1c00629)
Supplement: Supplementary file 1 — jp1c00629_si_001.pdf [file jp1c00629_si_001.pdf]

Supporting Information for

Twisted Intramolecular Charge Transfer (TICT) Controlled by Dimerization: An Overlooked Piece of the TICT Puzzle

Ahmed M. El-Zohry[a,b,*], Esam A. Orabi[c,*], Martin Karlsson[d] and Burkhard Zietz[a]

[a] *Department of Chemistry – Ångström Laboratory, Uppsala University, Box 523, SE-751 20 Uppsala, Sweden.*
[b] *Department of Physics – AlbaNova Universitetscentrum, Stockholm University, SE-10691 Stockholm, Sweden.*
[c] *Department of Chemistry, University of Manitoba, Winnipeg, Manitoba R3T 2N2, Canada*
[d] *Applied Physical Chemistry, KTH Royal Institute of Technology, Teknikringen 30, SE-10044 Stockholm, Sweden.*
*Ahmed M. El-Zohry (amfzohry@yahoo.com, ahmed.elzohry@fysik.su.se), or
*Esam A. Orabi orabiesam@gmail.com.

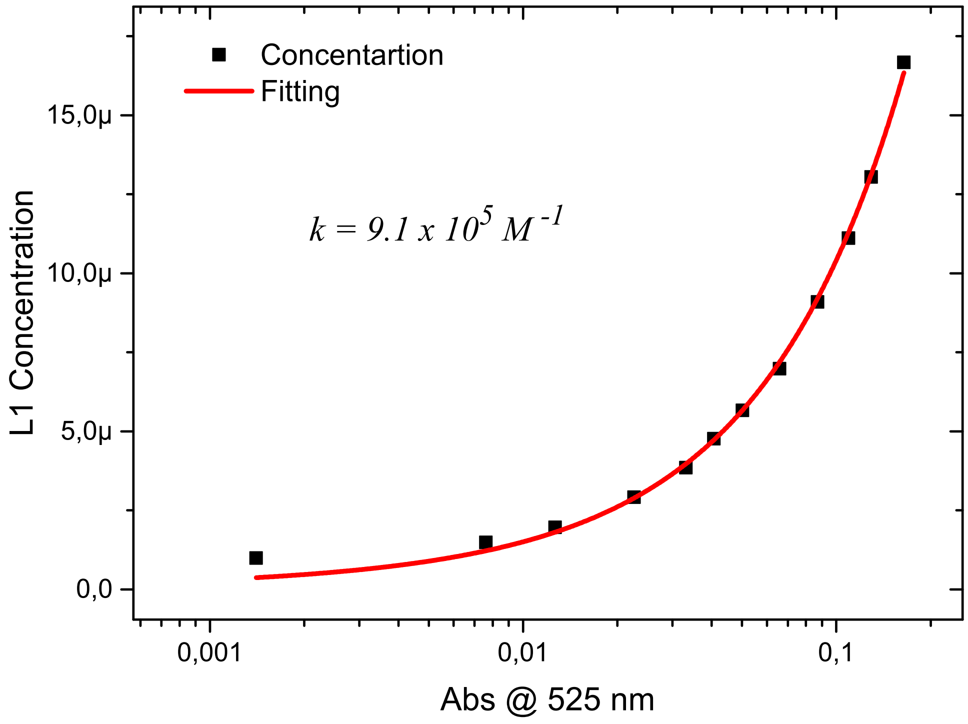

Figure S 1: Benesi-Hildebrand fitting equation for absorption changes for L1 in MeCN at 525 nm. The titration with different bases indicates, at least, a deprotonation constant of L1 in MeCN is ca. $1e^{-15}$.

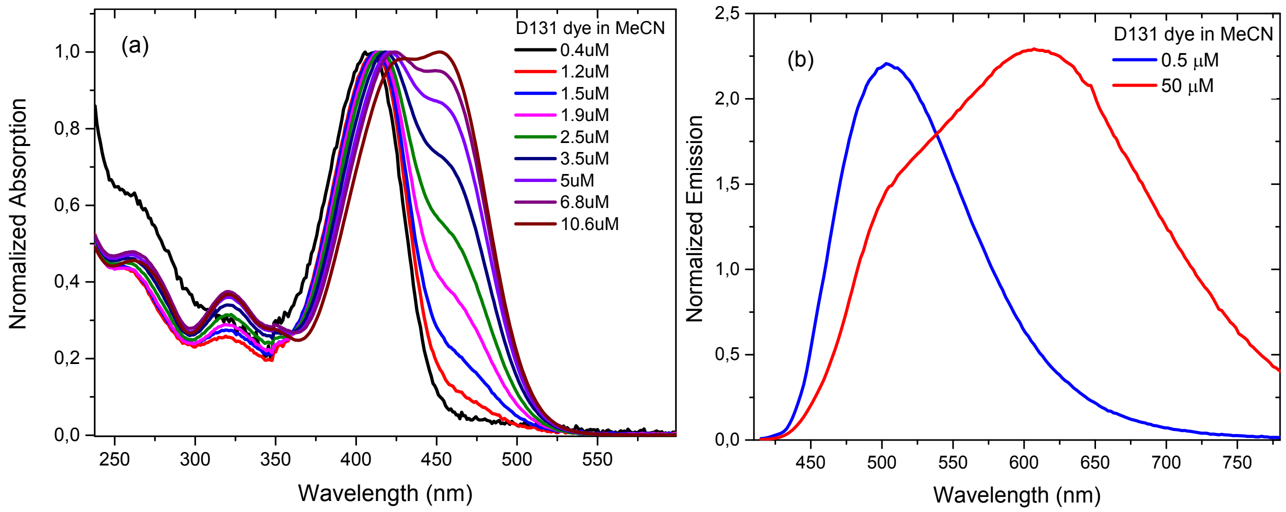

Figure S 2: Normalized absorption (a) and emission (excitation 400 nm) for cyanoacrylic dye D131 at low and high concentration in MeCN, showing two distinct emission bands (b).

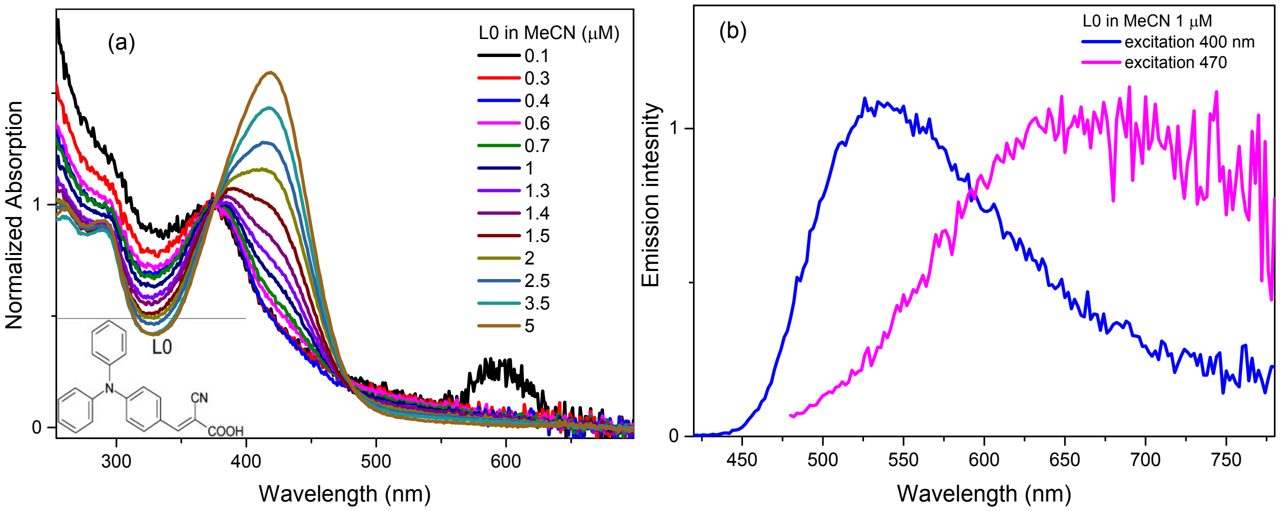

Figure S 3: Normalized absorption (a) and emission (b) spectra for L0 in MeCN at different concentrations and excitations wavelengths.

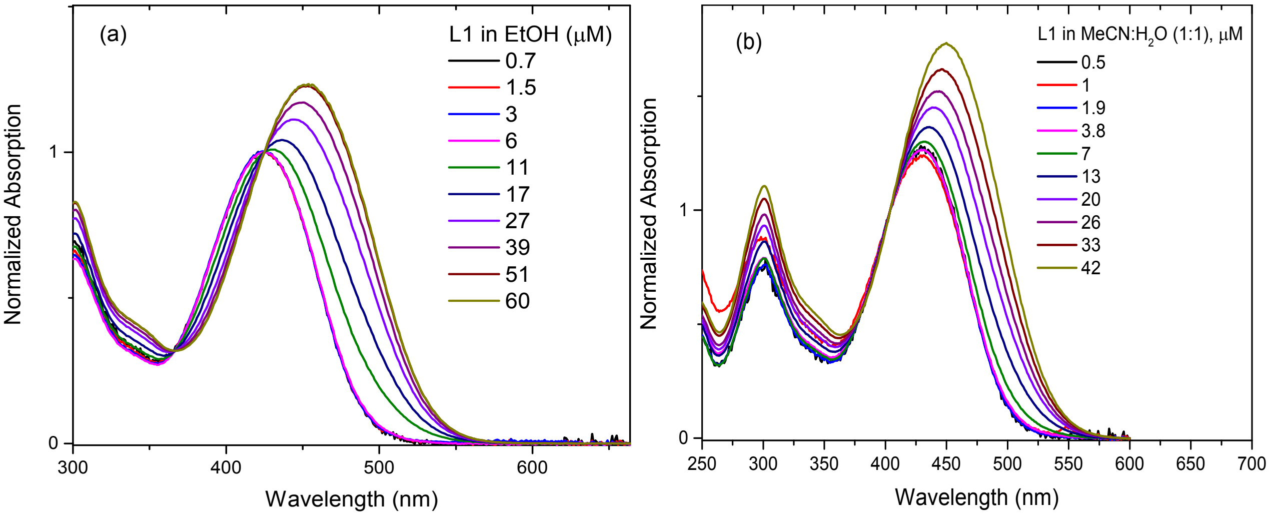

Figure S 4: Normalized absorption of L1 dye in EtOH (a), and in MeCN:H_2O (1:1) (b), upon changing concentrations.

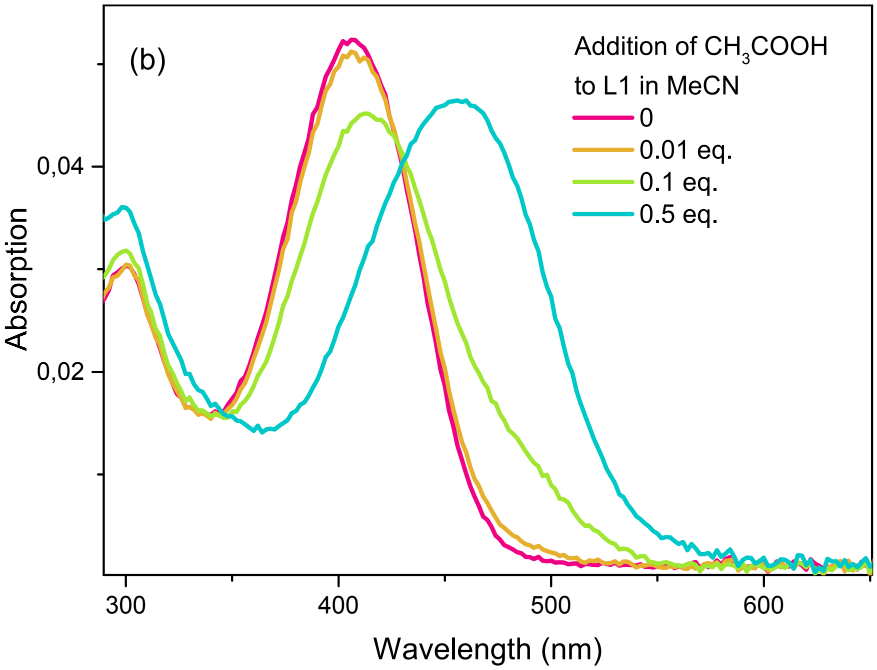

Figure S 5: Addition of different concentration of acetic acid to monomer form of L1 in MeCN.

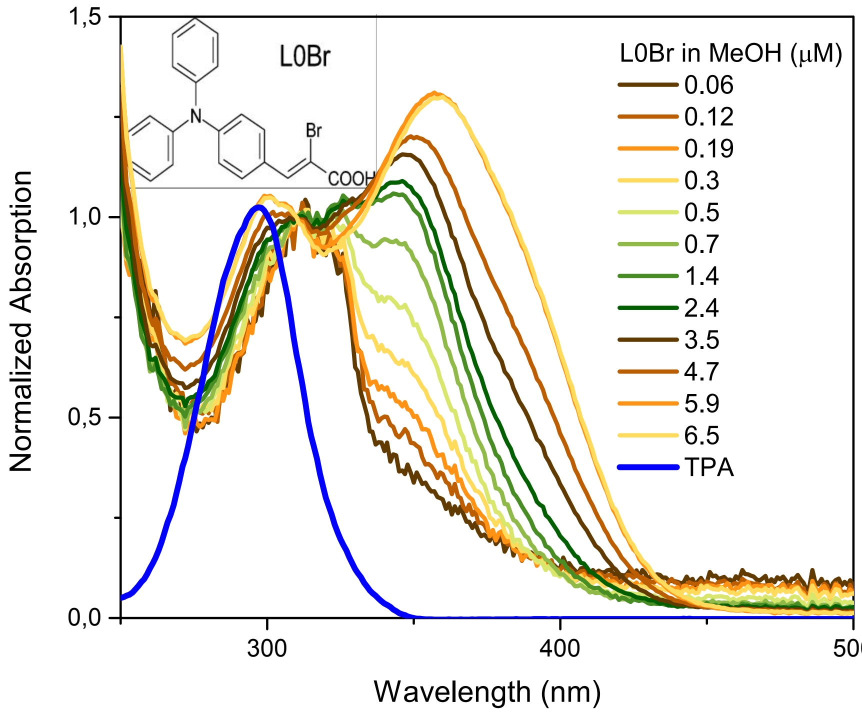

Figure S 6: (a) Normalized absorption for various concentration of L0Br in MeOH, along with the absorption of the donor triphenyl amine moiety in blue color. (b) Addition of various CH_3COOH concentrations to the monomer form of L1 in MeCN.

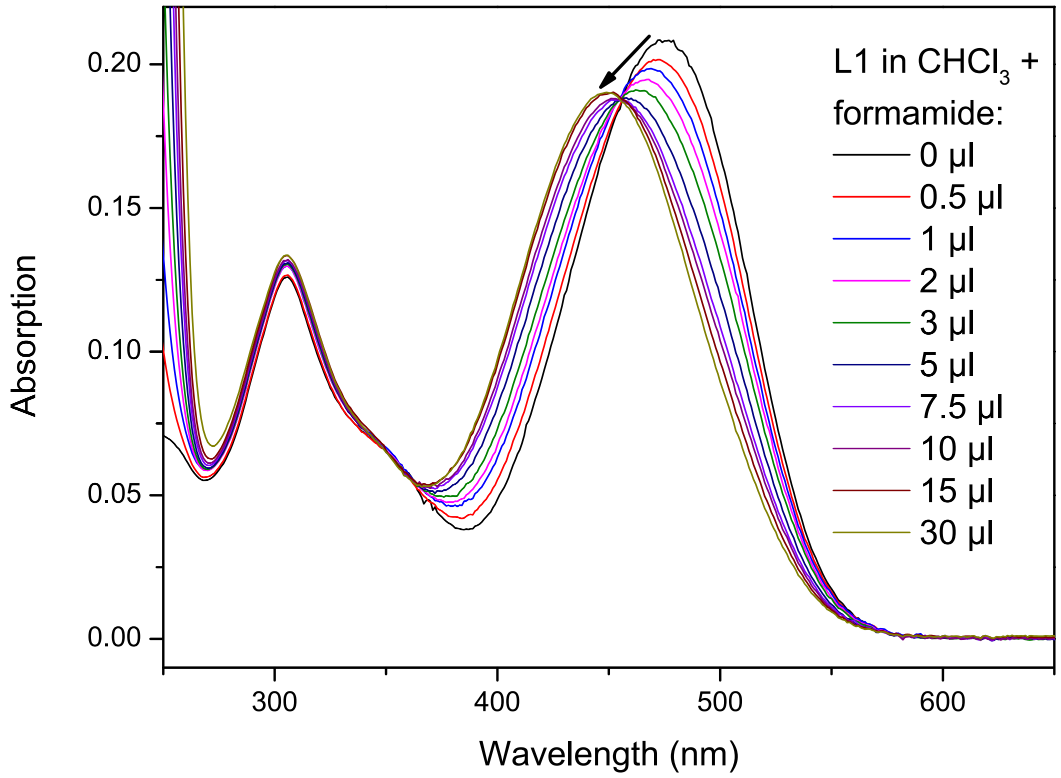

Figure S7: Titration of L1 in $CHCl_3$ with formamide. The isosbestic point around 455 nm points to a clear conversion between two species, not a gradual shift due to changes in solvent properties such as polarity.

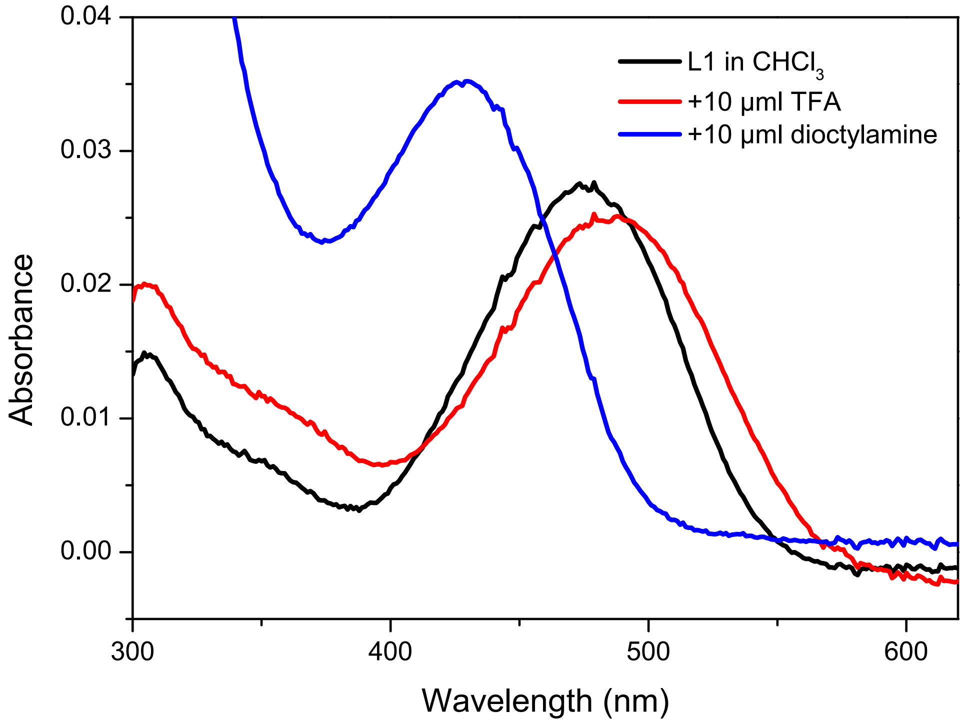

Figure S8: Addition of TFA to L1 in $CHCl_3$; protonation red-shifts the absorption, giving spectra similar to L1 dimers, whereas dioctylamine deprotonates and gives the monomer form.

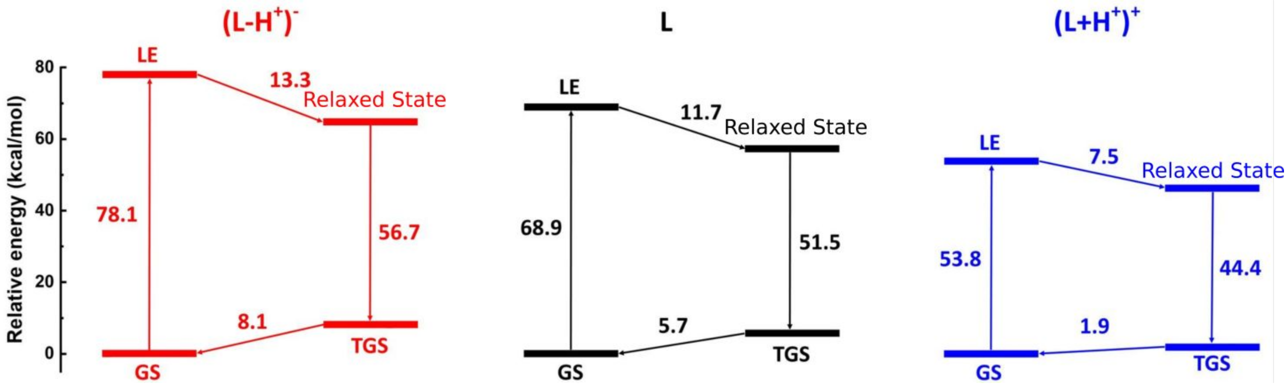

Figure S 9: Comparing between energy levels for the deprotonated, neutral, and protonated form in the GS (ground state), LE (locally excited state), Relaxed state in the excited state, and TGS (thermally excited state).

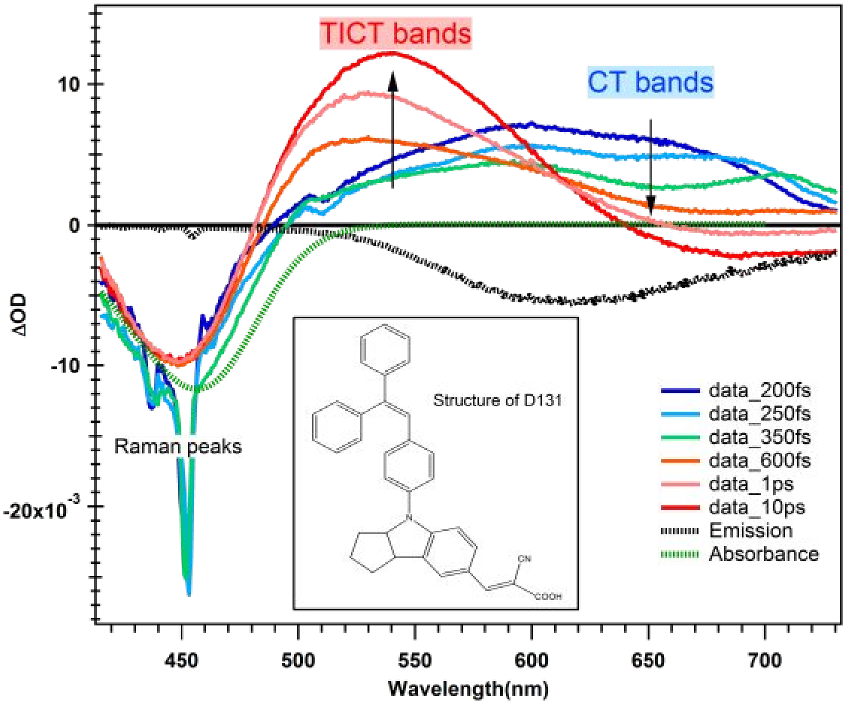

Figure S 10: The spectral change of D131 in MeCN over time. The structure of D131 is shown in the inset. Steady state measurements, absorbance, and emission, are shown in dotted lines.
